# Screening Bioremediation for the Effective Removal of Regulated and Emerging Contaminants from Mining Wastewater

**DOI:** 10.3390/molecules31091494

**Published:** 2026-04-30

**Authors:** Niroshan Gajendra, Anamaria Iulia Török, Deniz Avsar, Mila Kristiina Pelkonen, Simion Bogdan Angyus, Ragne Lundeby Grønvold, Claudiu Tănăselia, Erika Andrea Levei, Laura Ferrando-Climent

**Affiliations:** 1 Department of Tracer Technology, Environmental Technology Section, Institute for Energy Technology, Instituttveien 18, 2007 Kjeller, Norway; deniz.avsar@ife.no (D.A.); laura.ferrando-climent@ife.no (L.F.-C.); 2 Research Institute for Analytical Instrumentation Subsidiary, National Institute of Research and Development for Optoelectronics, Donath 67, 400293 Cluj-Napoca, Romania; bogdan.angyus@icia.ro (S.B.A.); claudiu.tanaselia@icia.ro (C.T.); erika.levei@icia.ro (E.A.L.); 3 Department of Environmental Safety and Radiation Protection, Environmental Technology Section, Institute for Energy Technology, Instituttveien 18, 2007 Kjeller, Norway; mila.pelkonen@ife.no (M.K.P.); ragne.gronvold@ife.no (R.L.G.)

**Keywords:** bioremediation, phytoremediation, phycoremediation, mining wastewater, toxic elements, emerging contaminants, screening experiments

## Abstract

Mining wastewater contains complex mixtures of regulated and emerging contaminants that challenge treatment technologies. This study evaluates the bioremediation potential of 10 phytoplankton species, including *Chlorella vulgaris*, and the aquatic fern *Salvinia natans* for removing contaminants from synthetic and mine outflow water. Batch screening experiments were conducted using synthetic wastewater containing regulated elements, rare earth elements (REEs), or selected organic flotation reagents, followed by validation using acidic mine outflow water from a decommissioned mine (Romania). All tested phytoplankton species and *Salvinia natans* showed high removal efficiencies for several priority elements, including Pb, Ag, Cr, Th, U, and multiple REEs. Organic flotation reagents were efficiently removed by all phytoplankton species. *Chlorella vulgaris* and *Salvinia natans* emerged as high-performing species and were further evaluated in mine outflow, where species-specific and matrix-dependent removal behavior was observed. Here, *Chlorella vulgaris* showed a higher average removal. Time-resolved analyses indicated a rapid initial removal followed by equilibrium phases, suggesting biosorption and bioaccumulation mechanisms. Li and Se showed limited removal capacities across all species. Photosynthetic pigment analysis revealed stress responses in *Salvinia natans* under acidic, multielement exposure. Overall, phycoremediation and phytoremediation represent effective low-chemical treatment strategies with potential for integration into a complementary mining wastewater treatment workflow.

## 1. Introduction

Bioremediation employs living organisms such as microbial communities, plants or their association to degrade, immobilize, or remove contaminants from wastewater through biologically mediated processes such as decomposition, precipitation, sorption, and accumulation [1,2,3]. These interconnected processes lead to contaminant adsorption onto the cell surface or within the biomass and subsequent transformation or degradation into less toxic forms. In addition to remediation of contaminated environments, bioremediation allows resource recovery and valorization of removed toxic elements [2]. Successful implementation of bioremediation requires selection of stress-tolerant, indigenous microorganisms and/or plants, together with a comprehensive understanding of synergistic interactions to ensure consortium resilience under field conditions [3].

Phytoremediation uses terrestrial or aquatic plant species as biological filters to accumulate, transform, and sequester toxic metals and organic contaminants [4]. Effective hyperaccumulator species are characterized by high bioaccumulation capacity and efficient translocation from roots to shoots. Phytoremediation pathways include phytovolatilization, phytodegradation, phytoextraction, and rhizofiltration through several mechanisms, including alkalization, complexation, precipitation, biotransformation, bioaccumulation, biosorption, and adsorption [5]. These processes are controlled by the presence of anionic functional groups—such as amide, amine, hydroxyl, carboxyl, sulfonate, and sulfhydryl groups—located on the cells’ surfaces, which facilitate metal binding, sequestration, and accumulation [5]. Despite its potential, phytoremediation presents several limitations including slow remediation rates, dependence on contaminant concentration and bioavailability, need of additional chelating agents to enhance uptake, and challenges in biomass disposal to prevent secondary pollution [6].

Phycoremediation uses the microalgal metabolic and biosorptive capacity for contaminant removal from wastewater. In addition, phycoremediation can include cyanobacteria (formerly known as “blue-green” algae), another group of phytoplankton. The advantages of phycoremediation include fast-growth rates, metabolic versatility, and tolerance of harsh conditions such as extreme pH and heavy metal concentrations that are lethal to other organisms [7,8,9]. Microalgae such as *Chlorella vulgaris*, *Chlamydomonas reinhardtii* and *Botryococcus braunii* as well as cyanobacteria such as *Synechococcus* sp. demonstrated suitable performances across diverse wastewater types [10,11]. Beyond removal of contaminants, the algal biomass enables resource recovery and value chain enhancement through products such as biofertilizers, biochar, animal feedstock, biofuels and electricity through microalgal fuel cells [8]. However, the harvesting of algal biomass is still challenging and more expensive than plant biomass harvesting due to the smaller cell size and dilute biomass concentrations [12]. Integrated remediation systems combining phycoremediation and phytoremediation can function synergistically in sequential, symbiotic, or spatially separated configurations to enhance treatment efficiency [13,14].

REEs are critical elements within contemporary geopolitical supply chains such as renewable energy technologies [15] yet threaten the aquatic ecosystems and human health by the large volumes of waters and contaminated effluents generated during beneficiation [16]. Bioremediation is a promising alternative for the management of these outflows in a sustainable manner. While wastewater treatment is being challenged with emerging contaminants such as pharmaceuticals, personal care products [17], and flotation reagents, nature-based solutions such as phytoremediation and phycoremediation provide sustainable pathways for removing complex pollutant mixtures. Water quality standards differ across geographic entities and regulatory frameworks, reflecting the high variety of contaminant groups. Although regulatory bodies such as the European Union have established limits for heavy metals and radioactive elements including uranium (U) and thorium (Th), critical elements such as lithium (Li) [18,19] and REEs [20] remain unregulated despite their demonstrated environmental toxicity. Furthermore, residuals of organic flotation reagents used in mineral beneficiation are not specifically regulated, yet were found to pose environmental risks [16]. The human health implications of these unregulated mineral beneficiation constituents are not fully understood.

With increasing interest in nature-based water treatments, comparative datasets that assess multiple aquatic taxonomic categories against both regulated and emerging mining-related contaminants under comparable conditions become important. Therefore, this study aims to (i) screen phytoplankton and an aquatic fern across multielement and organic-reagent matrices, (ii) identify best-performing candidates for validation in an environmental mine outflow matrix, and (iii) use time-resolved profiles to infer practical treatment windows and constraints. This work specifically investigates the efficiency of these bioremediation approaches for decontaminating REE beneficiation effluents through a series of experiments designed to identify, characterize, and compare suitable biological taxa across both phytoremediation and phycoremediation methods.

## 2. Results

### 2.1. Screening Experiments with Synthetic Wastewater

#### 2.1.1. Regulated and Unregulated Elements

**Figure 1:**
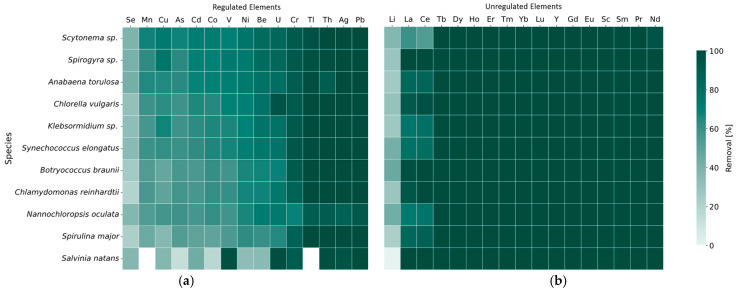
Removal efficiency (%) after 7-day exposure of phytoplankton species and *Salvinia natans* to synthetic wastewater containing elements at an initial concentration of 50 µg/L: (**a**) regulated elements; (**b**) unregulated elements.

Removal efficiencies after 7 days of exposure to synthetic wastewater containing regulated elements at an initial concentration of 50 µg/L were comparable for all tested phytoplankton species (Figure 1a). For most elements, the removal efficiencies exceeded 50%, with the highest values (>80%) observed for Pb, Ag, Th, Tl and Cr, and the lowest (<50%) for Se (Tabel S1). *Salvinia natans* have removal efficiencies comparable to those of the phytoplankton species, ranging from 70% to 90% for V, Cr, Ag, Cd, Pb, and U and below 60% for Co, Ni, Cu, As, and Se (Tabel S1).

All phytoplankton species showed almost full removal of the majority of unregulated elements with the exceptions of Li, La, and Ce, from an initial concentration of 50 µg/L (Figure 1b). The phytoplankton species with the highest removal efficiency across all unregulated elements were *Chlorella vulgaris*, *Spirogya* sp., and *Botryococcus braunii*. The removal efficiencies by *Salvinia natans* followed a similar pattern to those observed for *Chlorella vulgaris*, *Spirogya* sp., and *Botryococcus braunii*. However, the Li removal efficiency of *Salvinia natans* was much lower (<15%) than that of the phytoplankton species (30–60%) (Table S1).

Taken together, these multielement screening results indicate broadly comparable removal performance across diverse phytoplankton species at low concentrations, while highlighting *Chlorella vulgaris* and *Salvina natans* as consistently high performers across many unregulated elements. Building on this strong inorganic removal capacity, the next subsection evaluates whether similar efficiencies extend to less toxic organic flotation reagents relevant for beneficiation process waters.

#### 2.1.2. Organic Flotation Reagents

**Figure 2:**
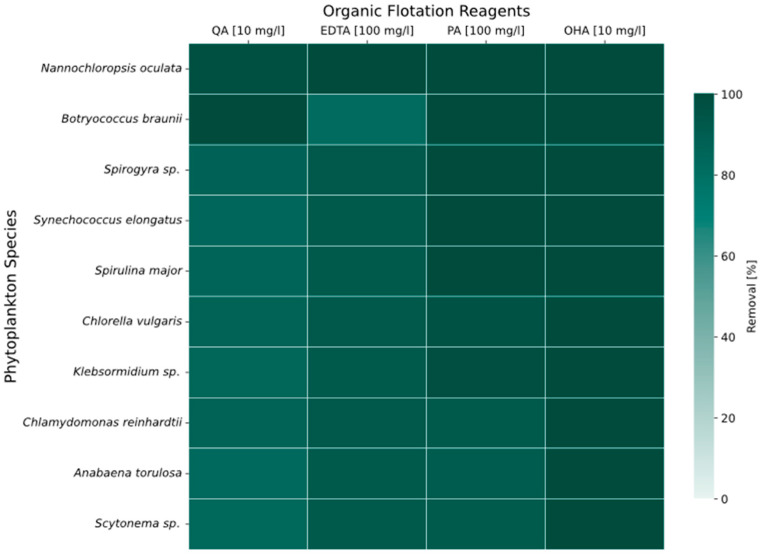
Removal efficiency (%) of quinic acid (QA), ethylenediaminetetraacetic acid (EDTA), phytic acid (PA), and octanohydroxamic acid (OHA) from synthetic wastewater containing 100 mg/L EDTA, 100 mg/L PA, 10 mg/L QA and 10 mg/L OHA by phytoplankton species.

All phytoplankton species achieved removal efficiencies of ≥80% of all studied organic flotation reagents (Figure 2). The highest removal efficiency was observed for octanohydroxamic acid (OHA) compared to quinic acid (QA), ethylenediaminetetraacetic acid (EDTA), or phytic acid (PA). The initial concentration of the four eco-friendly organic flotation agents were adjusted to their analytical detection limits: 100 mg/L for EDTA and PA, and 10 mg/L for QA and OHA. Consequently, phytoplankton effectively removed higher absolute concentrations of EDTA and PA. To test the robustness of these findings beyond simplified water chemistry, the subsequent section validates performance using a complex, environmentally derived mine outflow.

### 2.2. Screening Experiments with Mining Wastewater

The outflow of the Apolo mine contains a variety of metals. Elevated concentrations were observed for Al, Mg, Fe, Zn, Mn, and Cu, all exceeding 100 µg/L. Pb with 100 µg/L and Cd with 20 µg/L exceeded their respective parametric values established by the EU Drinking Water Directive (Table 1). In contrast, U and Tl are below 10 µg/L.

**Figure 3:**
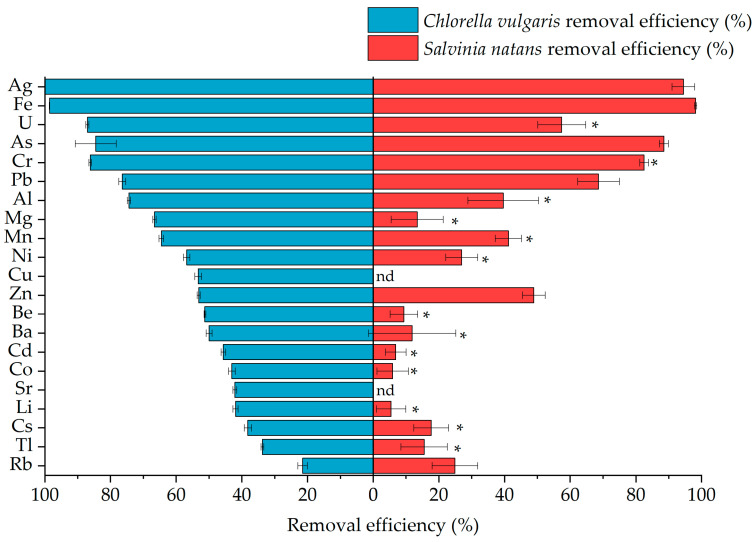
Removal efficiencies of *Chlorella vulgaris* and *Salvinia natans* after a 7-day exposure period to mine outflow. The asterisk (*) indicates significant differences (*p* ≤ 0.05) between the removal efficiency of *Chlorella vulgaris* and *Salvinia natans* for the same element, as determined by Tukey’s HSD post hoc test.

*Chlorella vulgaris* showed high removal efficiencies (>80%) for Ag, Fe, U, Cr, and As from the mine outflow, while *Salvinia natans* removed 60–80% of Ag, Fe, Cr, and As (Figure 3). Moreover, *Chlorella vulgaris* showed significantly greater removal efficiencies than *Salvinia natans* for Li, Be, Mg, Al, and Cd, while no significant differences between the species were observed for the removal of Ag, As, Fe, Pb, Rb, and Zn (Figure 3). Overall, the real case matrix experiment confirms high removal efficiency of several key constituents while revealing stronger species-dependent differences than those observed in synthetic water, consistent with the higher chemical complexity and broader concentration range of the mine outflow. To link removal performance with uptake and physiological effects, the following subsection quantifies elemental accumulation in *Salvinia natans* biomass and associated responses.

### 2.3. Element Accumulation in Salvinia natans and Plant Response

**Figure 4:**
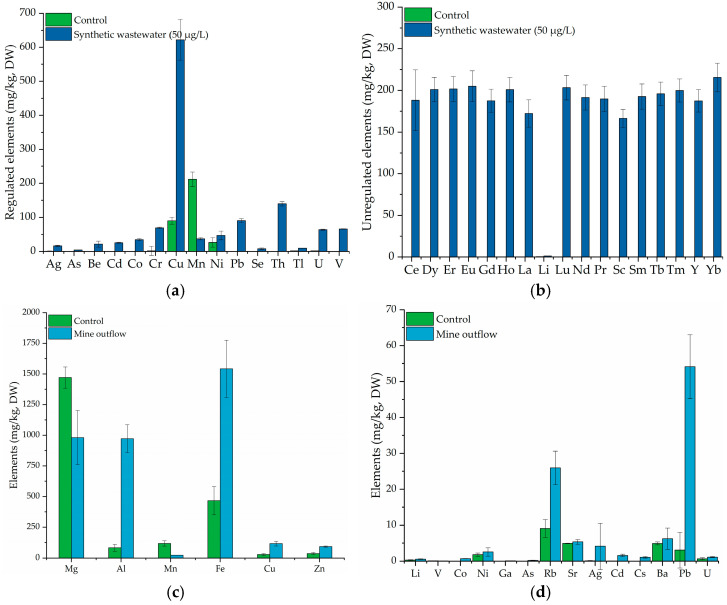
Concentration of elements in *Salvinia natans* biomass (blue) after exposure to (**a**) synthetic wastewater containing regulated elements, (**b**) synthetic wastewater containing unregulated elements, (**c**) mine outflow for major elements (Mg, Al, Mn, Fe, Cu, Zn), (**d**) mine outflow for minor elements (Li, V, Co, Ni, Ga, As, Rb, Sr, Ag, Cd, Cs, Ba, Pb, U) in comparison to the *Salvinia natans* control group (green) that was exposed to Hoagland nutrient solution.

*Salvinia natans* biomass exhibited higher elemental concentrations when exposed to toxic elements from synthetic wastewater compared to the control group, demonstrating effective uptake (Figure 4). Control plants contained relatively low elemental concentrations, reflecting background levels from the uncontaminated nutrient solution.

Several toxic elements showed substantial increases in *Salvinia natans* following exposure, including Ag (16.5 mg/kg DW), Cr (69.2 mg/kg DW), Cd (25.5 mg/kg DW), Pb (90.0 mg/kg DW), and U (63.9 mg/kg DW). A notable accumulation was also observed for unregulated elements, such as Ce, Nd, Sm, and Yb, indicating a strong affinity of *Salvinia natans* for these emerging contaminants (Figure 4b). The concentrations of Al, Fe, and Zn in *Salvinia natans* samples increased significantly following exposure to mine outflow compared to the control group. In contrast, Mn concentrations were significantly reduced in treated plants relative to the controls (Figure 4c). The results further demonstrate that *Salvinia natans* possesses a considerable capacity for the accumulation of Rb, Pb, Cd, and As (Figure 4d). No statistically significant differences were detected in Mg or Cu concentrations between treated and control plants, suggesting possible competitive interactions during the uptake process.

**Figure 5:**
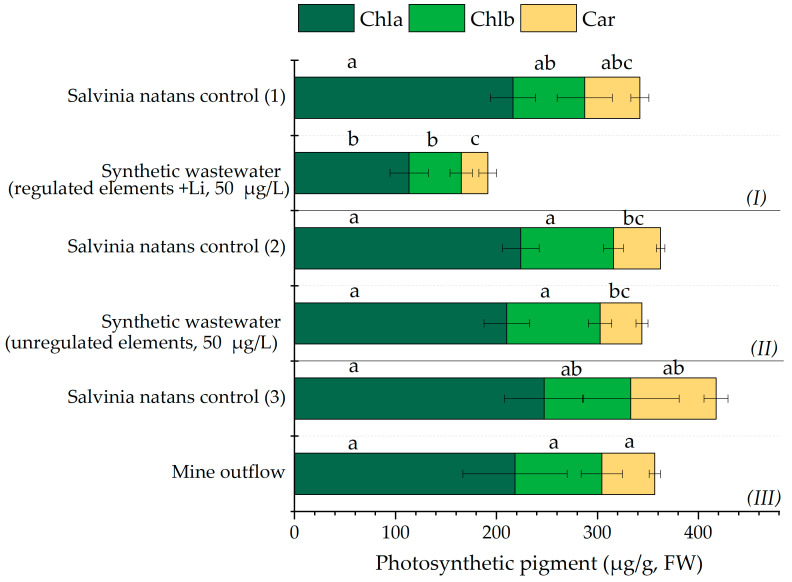
Photosynthetic pigment content: chlorophyll a (Chla), chlorophyll b (Chlb) and carotenoids (Car) (mean ± SD, n = 3 for the independent experiments) after the 7 days of exposure to regulated (***I***) and unregulated elements from synthetic wastewater (***II***) and to mine outflow (***III***) in comparison with their control. Different letters indicate significant differences (*p* ≤ 0.05) among groups tested by Tukey-HSD post hoc test.

The stress response of *Salvinia natans* to elemental exposure is reflected in its photosynthetic pigment levels (Figure 5). Plants exposed to regulated elements showed a significant decrease in chlorophyll a (Chl a) and carotenoid (Car) content, while chlorophyll b (Chl b) remained unchanged (Figure 5). When exposed to unregulated elements, *Salvinia natans* exhibited no significant changes in photosynthetic pigment concentrations, suggesting a relatively high tolerance or effective detoxification capacity under the tested conditions.

### 2.4. Temporal Variability of Element Concentrations in Synthetic Wastewater

The temporal variations of elemental concentrations in synthetic water treated by *Chlorella vulgaris* and *Salvinia natans* are presented in Figure S1. A notable decrease in the concentrations of most elements was observed within the initial 1–3 h of contact time, indicating rapid initial uptake and removal. A second pronounced decrease occurred after 24 h, suggesting a secondary uptake phase. Among the studied elements, Cu exhibited higher variability and remained at elevated concentrations in the synthetic solution for a longer period than the other elements. In contrast, elements such as Cd, Pb, V, and Cr showed a more gradual decrease over time. Li and Se represent limitations in removal throughout the entire observation period, while U and Nd were rapidly removed by the majority of species.

**Figure 6:**
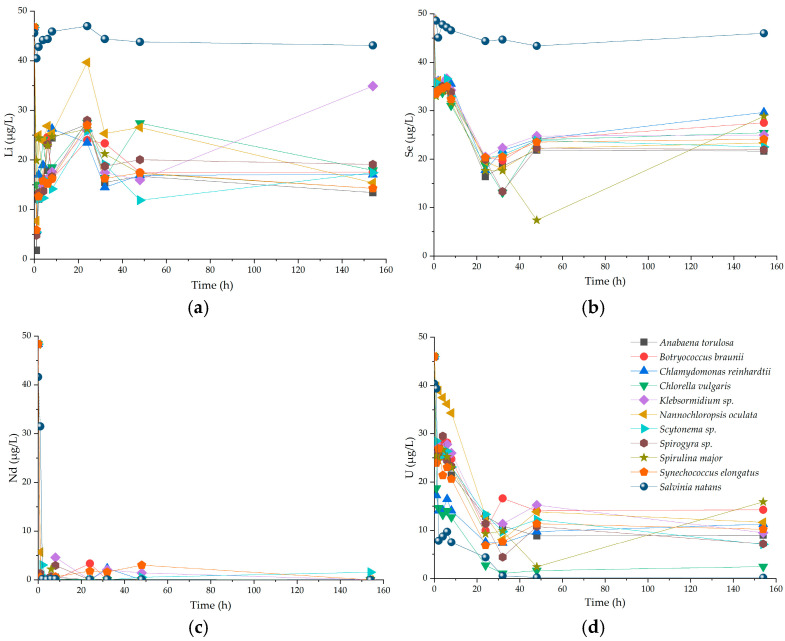
Temporal variation of element concentrations in water samples treated with exposed species (phytoplankton and *Salvinia natans*) during a 7-day exposure to regulated and unregulated elements: (**a**) Li removal; (**b**) Se removal; (**c**) Nd removal; and (**d**) U removal.

The Li removal by phytoplankton (Figure 6a) varied across species and incubation time. The highest Li removal occurred within the first 12 h, followed by a partial increase and stabilization of Li concentrations after 48 h. *Anabaena torulosa* was the only species that removed Li down to 2 µg/L after only 1 h. In contrast, *Salvinia natans* did not significantly change Li concentrations throughout the entire incubation time. Regarding Se, all phytoplankton species and *Salvinia natans* showed the lowest removal efficiencies among all regulated elements (Figure 6b). Most phytoplankton species showed peak Se removal around 24 h, followed by a gradual increase and plateau after 40 h, reaching around 25% removal efficiency. *Spirullina major* showed the highest Se removal efficiency at 44 h of incubation. *Salvinia natans* did not show significant changes in Se concentration between initial and final time points. Nd is an example of a rapidly removed REE by both phytoplankton and *Salvinia natans* (Figure 6c). All tested species removed nearly the entire initial Nd concentration within the first sampling time point. Furthermore, Nd concentrations remained relatively stable throughout the entire incubation period, showing only minor variations across all species. The U concentrations (Figure 6d) show differences between phytoplankton species across nine time points during the 7 days of incubation. All phytoplankton species removed at least 50% of the initial U concentrations. *Chlorella vulgaris* exhibited the highest U removal, completely removing the added U within the first 24 h. In addition, the 12 h day and night cycles show variations across the species, especially after the first 24 h.

**Figure 7:**
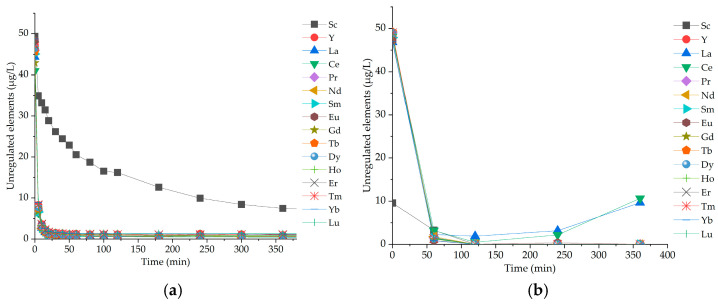
Temporal variation of element concentrations in *Salvinia natans* (**a**) and *Chlorella vulgaris* (**b**) exposed to unregulated elements.

The time-dependent removal of regulated elements by *Salvinia natans* indicated rapid adsorption, with faster uptake observed for unregulated elements than for the regulated ones. Adsorption of V, Cr, Ag, Cd, Pb, and U was the most rapid among regulated elements, reaching equilibrium within 3–4 h, whereas unregulated elements reached equilibrium in less than 3 h (Figure 7a). *Chlorella vulgaris* removed REEs efficiently within 60 min. While most REEs remained at equilibrium thereafter, Ce and La showed a subsequent increase, with a peak observed at the 360 minute mark (Figure 7b).

## 3. Discussion

### 3.1. Implication from Synthetic Wastewater Experiments

The synthetic wastewater experiments showed broad removal capacities by phycoremediation and phytoremediation. All 10 tested phytoplankton species and the aquatic fern showed their best removal efficiencies towards REEs, Pb, Ag, Th and Tl collectively. The synthetic wastewater mimics realistic multi-contamination systems and goes beyond the current literature. There is a need for screening under realistic wastewater conditions to properly evaluate bioremediation performance and its limitations in industrial and mining wastewater contexts.

Among the phytoplankton species, *Chlorella vulgaris*, *Spirogyas* sp., and *Botryococcus braunii* were the best-performing, achieving removal efficiencies comparable to *Salvinia natans*. However, removal efficiencies did not vary significantly among the phytoplankton species for a given element. Notably, while nine of the phytoplankton species are freshwater species, the saltwater species *Nannochloropsis oculata* exhibited removal efficiencies comparable to those of its freshwater counterparts. Overall, these findings suggest that the tested species display element-specific behavior when exposed to a multielement mixture of elements at low concentration (50 µg/L) and acidic pH. From a temporal perspective, greater variability was observed at earlier time points and among species. In most cases, the maximum removal efficiencies were achieved after 48 h and remained relatively constant thereafter. The observed removal trends confirm the potential of bioremediation for regulated elements, offer insight into the bioremediation of unregulated and emerging pollutants, and highlight its limitations.

Pb is a well-regulated element across geographical regulatory bodies such as WHO, EU, and the EPA for instance. Its removal through bioremediation using a variety of organisms has been well documented [21,22]. In this context, the present study reinforces that Pb is among the most efficiently removed elements in multielement solutions, even in the presence of competing regulated and toxic elements at low pH (Figure 1a). The observed removal efficiencies (>90%) across species are consistent with previously reported values for both multi- and monoelement incubation studies, including those conducted at Pb concentrations of up to 10 mg/L [23]. On the other hand, Ag is addressed by the WHO and Canadian regulatory water framework but not by the EU and the EPA. The toxicity of Ag, especially in aquatic environments, remains insufficiently understood. Ag is primarily introduced through anthropogenic activities and often occurs as nanoparticles (e.g., Ag_2_O, Ag_2_S, AgCl_x_), which can transform into ionic Ag (Ag^+^). This transformation enables Ag to participate in a wide range of reaction pathways in aquatic environments [24]. Even though Ag has been investigated in bioremediation studies [25], it is not as widely in focus as Pb, Cd or As. In the present artificial wastewater experiment, Ag was removed with comparable efficiency to Pb by all tested species under competitive conditions with other regulated elements (Figure 1a). Tl is a known toxic element that originates from both natural sources, such as mineral ores and volcanic activities, and anthropogenic sources, such as coal combustion and cement production [26,27]. Tl toxicity is reported to exceed that of Hg for instance. Interestingly, while Tl is regulated in the US (EPA; MCL = 2 µg/L) due to its toxicity, Tl is not yet regulated in the EU drinking water framework. High Tl removal efficiencies have been reported in previous studies, showing that *Chlorella vulgaris* is able to remove 100% Tl in concentration ranges ≤150 mg/L [28]. The results of the present experiment demonstrate significant Tl targeted removal by a variety of species under multielement competitive conditions (Figure 1a).

U and Th are naturally occurring radioactive elements and therefore are widely the focus of bioremediation studies [29]. Since U and Th commonly occur in mineral ores, their remobilization and subsequent release into the environment are of particular concern [30]. Previous studies have demonstrated that bioremediation can provide efficient U and Th removal from aquatic environments [29,31]. *Chlorella vulgaris* has been shown to remove up to 90% of U at concentrations exceeding 20 mg/L [32] and up to 99% of Th at concentrations of 50 mg/L [31]. The present study showed consistently high removal of Th by all tested species in multielement wastewater (>90%). Regarding U, *Chlorella vulgaris* and *Salvina natans* showed significantly higher removal efficiencies (>90%; Figure 1a) and faster uptake (<40 h; Figure 6d) compared to the other tested species. Hence, *Chlorella* *vulgaris* and *Salvina natans* were selected for further experiments using real wastewater matrices (mine outflow). However, it is worth noting that the adsorption behavior of these radionuclides may vary depending on the prevailing geochemical conditions and ion speciation, solution pH being of particular importance. Previous studies have shown that Th sorption by *Chlorella vulgaris* is highest at pH 4, where Th likely occurs as hydrolyzed species such as Th(OH)_2_^2+^, whereas at higher pH values, the formation of more complex hydrolyzed species may decrease biosorption [33]. Similarly, U mobility and bioavailability are tightly bound to its speciation. Under acidic conditions, positively charged uranyl species predominate, while the increasing pH may shift the speciation towards stable and mobile U-carbonate complexes, potentially influencing the sorption behavior [32]. Additionally, remobilization of previously retained U has been observed during incubation with living *Chlorella* cultures, whereas this effect is not reported at higher U concentrations that induce cell death [32].

REEs are classified as critical raw material and currently are not regulated in drinking water, but are considered potential emerging contaminants if released uncontrolled into the environment [16,34]. The biosorption of REEs sparks interest not only in terms of bioremediation but also in regards to beneficiation in REE ore processing [35]. The screening experiments in this study showed that all REEs were significantly removed by all tested species (Figure 1), with most being almost entirely removed within the first hours (Figure 6c, Nd as an example). Previous studies demonstrated the effective biosorption of specific REEs by phytoplankton, such as Nd biosorption by *Chlorella vulgaris* (>100 mg/g biomass) [35,36]. The present study provides new insights into multielement REE removal from wastewater, showing high removal efficiencies (>95%; Figure 1) across all tested species and REEs, with the exception of Ce and La. To the best of our knowledge, this is the first study to investigate REE removal using such a diversity of conditions in *Salvinia natans* which exhibited the best overall performance. A more detailed analysis of REE removal trends revealed that Ce and La showed lower removal efficiencies than the heavier REEs in certain phytoplankton species (ranging from 60 to 99%; Figure 1). In addition, the removal kinetics of Ce and La were slower and more variable in the phytoplankton treatment experiments (Figure 7b). This observation is consistent with findings in previous REE-based algal studies [37]. On the one hand, La and Ce have larger ionic radii than other REE ions, which may result in weaker surface complexation [38]. Furthermore, the comparable ionic radius of La^3+^ to Ca^2+^, combined with its affinity for oxygen-donor ligands, may result in interactions with Ca-binding sites and distinct biological uptake pathways [39,40]. On the other hand, Ce undergoes specific redox cycling (Ce^3+^/Ce^4+^) that also affects its bioaccumulation in aquatic environments [41]. These chemical features of La and Ce might explain the transient decrease in removal efficiency observed during the first day of incubation (Figure 7b), likely reflecting dynamic speciation changes and partial desorption processes.

The main limitations identified in the screening experiments were Se (removal efficiencies < 41%), among the regulated elements, and Li (removal efficiencies < 45%), among the emerging and unregulated elements. Se is an essential trace element for algae but toxic in elevated concentrations. Higher Se removal efficiencies (>80%) under similar syntheticwastewater conditions have been reported for adsorbents such as MOF, biochar, magnetite, and activated carbon (Avsar et al., in prep. [42]). Li can be considered an emerging contaminant, commonly released from sources such as battery waste. Previous studies [18,43] have shown that the Li uptake of *Salvinia natans* and *Chlorella vulgaris* is limited compared to other elements, such as Rb, and it was attributed to its toxic effects [44]. Xu et al., 2024, reported that low Li^+^ concentrations (as low as 1 µg/L) can induce hormetic effects in the phytoplankton species *C. zofingiensis*, whereas higher concentrations (50 mg/L) exhibit toxic effects [45]. Other incubation experiments using the phytoplankton species *Oocystis solitaria* revealed that pH, Li concentration, and temperature can influence Li removal [46]. While many of these studies used Li concentrations of up to 50 mg/L, even at 50 µg/L concentration in an REE-containing matrix, Li removal efficiencies remained low, ranging between 20 and 45% across all tested species (Figure 1b). In the mine outflow water Li concentration was 12 µg/L and *Chlorella vulgaris* and *Salvina natans* showed removal efficiencies similar to those observed in synthetic wastewater. However, *Chlorella vulgaris* exhibited higher removal efficiencies (42%) compared to *Salvina natans* (5%).

Thus, synthetic multielement screening serves as an effective initial method to identify taxa and groups of pollutants that can be efficiently addressed through bioremediation. It also highlights more challenging contaminants, such as Se and Li, which may need additional treatment strategies.

### 3.2. Implication from Mining Wastewater Experiments

The Apolo mine outflow was used as the real wastewater matrix to evaluate the best-performing species, *Chlorella vulgaris* and *Salvinia natans*. The synthetic wastewater had a similar acidic pH range but consisted of a simplified matrix based on distilled water and a defined multielement composition (50 µg/L per element). Tests conducted using mine outflow involved a more diverse elemental composition, spanning several orders of magnitude in concentration (Table 1). This increased compositional diversity reflects the strong element specific removal efficiencies of *Chlorella vulgaris* and *Salvinia natans*. Similar trends in both synthetic and mine outflow screening experiments were observed, with high selectivity and removal efficiencies for Ag, Pb, and U (Figure 3). In contrast, removal efficiencies of Tl and As show inverted trends between synthetic wastewater and mine outflow. A significantly higher As removal was observed in the mine outflow for both *Chlorella vulgaris* and *Salvinia natans*, likely due to the roughly 35 times lower As concentrations (1.4 µg/L) than in the synthetic wastewater (50 µg/L). By contrast, Tl removal was substantially lower in the mine outflow (<40%) compared to nearly complete removal (>95%) in the synthetic wastewater, despite the Tl concentration in the mine outflow (2.82 µg/L) being approximately 25-fold lower than in the synthetic wastewater. These observations indicate that removal efficiency is not solely governed by initial concentration. For example, Fe was almost entirely removed by *Chlorella vulgaris* and *Salvinia natans*, despite its high concentration in the mine outflow (6096 µg/L). Similarly, *Chlorella vulgaris* showed substantial removal of Mg (8201 µg/L) and Al (16675 µg/L), which were the two most abundant elements in the mine outflow (Table 1). In the case of Fe, *Chlorella* *vulgaris* has removed Fe at concentrations of 25 mg/L in previous mine outflow studies almost entirely, showing that this study did not reach the limit of *Chlorella vulgaris* removal capacity [47]. Aquatic macrophytes have a great phytoremediation potential, as shown by *Pistia stratiotes* and *Eichhornia crassipes*, which achieved removal efficiencies of >80% for Fe and >70% for Mn after exposure to mixed wastewaters derived from acid mine drainage, coal mining (Jambi Province), and domestic sources (Lampung, Indonesia) [48]. Additionally, they showed high removal efficiency for other pollutant parameters such as biological oxygen demand (up to 98%), chemical oxygen demand (up to 99%), phosphate (up to 73%), ammonia (up to 70%), nitrite (up to 48%), and nitrate (up to 91%). However, other metal contaminants were not reported which can influence the Fe and Mn removal efficiencies [48].

Hence, the contrasting trends in As and Tl removal are likely linked to site-specific matrix effects, such as the availability of anions and organics, and the presence of competing cations. In general, natural and lab-based systems may additionally differ in terms of pH, light intensity, and nutrient supply [25]. These mechanisms, however, were beyond the scope of the present study. The differences between synthetic and real wastewater show that treatment results from simplified matrices cannot directly be extrapolated to environmental conditions, especially for elements affected by speciation and competition. These findings highlight the need to separately assess organic process chemicals, as their transformation and by-products may further influence treatment performance and environmental safety.

### 3.3. Removal of Organic Substances

The screening experiments, in which all phytoplankton species were exposed to less toxic and more sustainable organic flotation reagents, resulted in high overall removal efficiencies (>80%; Figure 2) across species and contaminants. This study shows a systematic and, to the best of our knowledge, novel cross-species screening that demonstrates that phytoplankton can achieve high removal efficiency for multiple eco-friendly organic reagents.

Therefore, these experiments indicate that phycoremediation is a promising approach for the treatment of wastewater with these flotation reagents, such as REE beneficiation effluents. Bioremediation is especially relevant for EDTA, which is not biodegradable [49]. While these results show quantitative reductions of the tested flotation chemicals at concentrations of 10 mg/L and 100 mg/L, respectively, this study did not investigate their potential degradation pathways and transformation products. The formation of such transformation products is more likely in complex process water matrices than in the ultrapure water matrix used in this study. A reduction in the parent compound concentration alone is insufficient to confirm a reduction in environmental risk. Thus, further screening experiments are needed to assess the potential environmental transformation products of the flotation agents and to evaluate the removal efficiency of total toxicologically relevant substances by phycoremediation.

### 3.4. Bioremediation Mechanisms

The comparison of screening experiments provides insights into the underlying bioremediation mechanisms. In the case of the *Salvinia natans*, only limited uptake was observed, as well as a possible competition with essential nutrients present in the growth medium (Figure 4). High removal efficiencies were observed for Al, Ag, Cr, Pb, and U, while moderate removal was observed for Fe, Cu, Ni, and Cd (Figure 1). Among the regulated elements, Cd, Pb, As, Cr, and Cu are of the greatest concerns due to their toxicity and the strict regulatory limits imposed on wastewater discharges [5,50]. The effective removal of these elements is especially relevant for industrial and mixed municipal effluents. The accumulation patterns suggest that removal is primarily governed by rhizofiltration and surface adsorption processes, facilitated by the strong binding affinity of metal ions to plant cell walls and extracellular polymers [51,52]. The variations in element accumulation in *Salvinia natans* can be attributed to element-specific behavior and competitive interactions among elements. Essential elements such as Zn, Fe, Mn and Cu are actively taken up via specific membrane transporters including zinc-regulated transporters (ZIP family proteins), iron-regulated transporter-like channels (IRT-like) for Zn and Mn, iron-regulated transporter 1 (IRT1) for Fe, and copper transporter (Ctr) for Cu. In contrast, non-essential or toxic metals, including Cd and Pb, can enter cells either passively or via transporters designed for essential metals (e.g., Cd competes with Zn for ZIP channels), while Cr is typically taken up via sulfate transporters. Overall, the uptake of non-essential metals occurs through pathways that have evolved for essential nutrients [53]. As is mainly taken up by plants through phosphate transporters, as well as via aquaporins or nodulin26-like intrinsic proteins (NIPs) located in root cells. Exposure to As typically leads to nutrient imbalances, reduced growth, and alterations in photosynthetic pigment contents [54,55], representing characteristic stress responses in aquatic plants.

In the case of unregulated elements, rapid interactions were observed upon contact with *Salvinia natans* and *Chlorella vulgaris* (Figure 7). The uptake/removal of REEs occurred very fast, within the first 1–2 h of exposure. In contrast to the other REEs, Sc exhibited a distinct behavior, showing slower and more gradual uptake. This difference can be explained by the smaller ionic radius and higher hydration energy Sc has compared to typical trivalent rare earth elements, resulting in a lower affinity for functional groups (carboxyl –COOH, phosphate –PO_4_, hydroxyl –OH) on the biomass surface [56]. Consequently, its interaction with binding sites is less favorable, leading to slower removal rates. The uptake of unregulated elements is strongly influenced by their proximity to the plasma membrane, where adsorption sites and transport proteins are located. Furthermore, the cation exchange capacity (CEC) of the biomass, particularly under acidic conditions, enhances the binding and subsequent uptake of these elements [57]. This suggests the potential for REE capture and recovery, offering opportunities for resource valorization. Overall, these findings highlight the high potential of *Salvinia natans* for the remediation of multielement contaminated wastewater, especially for trace metals and radionuclides that are often insufficiently addressed by conventional treatment processes. The screening experiments indicated a limited capacity of *Salvinia natans* for Li removal. A possible explanation could be the competitive ion interactions at cell surface and membrane transport level. Saturation of negatively charged surface binding sites of the biomass by divalent cations such as Ca^2+^ and Mg^2+^ likely restricts Li^+^ adsorption, blocks transport towards the plasma membrane, resulting in a pronounced Ca–Li antagonism. Furthermore, Li^+^ uptake in plants is mediated by non-specific cation transport systems, which are typically involved in K^+^ transport [58]. However, Li uptake can be inhibited under elevated K^+^ concentrations due to the competition for shared transporters. In line with the observation of Török et al. (2022) [18], elevated concentrations of Ca^2+^, Na^+^, Mg^2+^, K^+^ can collectively suppress Li accumulation in *Salvinia natans*. This suppression is attributed to competition for cell wall binding sites and non-specific cation transporters, that ultimately reduce Li^+^ mobility, adsorption, and membrane transport.

Exposure of *Salvinia natans* to synthetic wastewater containing regulated (+Li) elements resulted in a decrease in chlorophyll a (Chla) content, indicating substantial physiological stress (Figure 5). A similar, though less pronounced, a decreasing trend was observed for chlorophyll b (Chlb) and total carotenoids (Car). Previously, it was reported that Li had a negative effect on chlorophyll content in plants ([59], with stronger effects typically observed in hydroponic systems compared to soil-based conditions. Following Li exposure, the Chla and Chlb contents were reduced by approximately 50% and 54%, respectively, which can be attributed to the rapid uptake of Li ions by plants, inducing inhibitory effects on chlorophyll biosynthesis and overall plant growth [59].

By contrast, exposure to unregulated elements or mine outflow had no significant effect on the photosynthetic pigments of *Salvinia natans*, despite similar initial concentrations. However, their bioavailability and physiological and/or biochemical impacts on the plant may differ. Metal-induced stress is known to interfere with chlorophyll biosynthesis, and to induce oxidative stress by reactive oxygen species (ROS), which can damage photosynthetic pigment molecules [60]. The presence of multiple toxic elements competing for the same transporters as essential micronutrients can enhance cellular accumulation and intensify stress responses even at low external concentrations. Chla was the most affected pigment in line with previous reports that stated that chlorophyll is rapidly degraded or its synthesis is inhibited under physiological stress conditions in aquatic mosses [60]. Similarly to our results, exposure to multi-metal synthetic wastewater (As, Cd, Cu, Pb, Zn) in *Azolla imbricata* aquatic species resulted in a significantly lower Chla content, reduced by 39–54% compared to the control group [61]. Previously, it was observed that *Myriophyllum aquaticum* plants exposed to mono-metal treatments (La, Ce, Nd, and Gd) for 10 days in high concentrations (50–500 mg/kg) showed a significant reduction in fresh weight in the case of La [62]. During Ce and Nd treatment, chlorophyll content decreased, while Gd had the strongest negative impact on the plants, causing chlorosis/necrosis [62]. Other studies have also reported that Gd can exert stronger toxic effects on photosynthetic pigments. Gjata et al. (2025) [63] reported significantly lower Chla and Chlb contents in *Lemna minor* after exposure to REEs in mono-element solutions, with a clear concentration- and time-dependent influence. Gd induced the most pronounced reduction in both pigments, whereas the effects of Ce and Nd were less pronounced [63]. In contrast, in the present study, synthetic wastewater containing REEs at 50 µg/L induced no significant changes in the photosynthetic pigment content of *Salvinia natans*, which indicates a dose-dependent response as well as a lower sensitivity of the species to REEs. The studied elements such as Ag, As, Cd, Co, Cr, Cu, Mn, Ni, Pb, Tl, U, and V, are well-known toxic elements that can interfere with chlorophyll biosynthesis, damage chloroplast membranes, disrupt electron transport, and induce oxidative stress, ultimately contributing to reduced photosynthetic pigment content and activity [64,65]. Thus, high removal of toxic elements may coincide with measurable physiological stress, reinforcing the need to jointly evaluate treatment efficiency and organism performance. To better contextualize treatment timeframe and distinguish between rapid and slow removal phases, the next subsection examines time-resolved concentration dynamics during exposure.

The mechanism of phycoremediation was not further investigated since the main aim of this study was to investigate the capacity of a variety of phytoplankton species towards a range of three different wastewater categories. However, comparison of the treatment and control experiments indicated physical changes, including loss of pigmentation and structural degradation of phytoplankton cells during treatment exposure. The relatively stable removal efficiencies despite these changes in phytoplankton morphology might indicate that biosorption is a key mechanism, as it can occur independently of cellular viability. Furthermore, the 12 h light and dark cycles, together with the results of the 24 h sampling point, suggest temporal shifts in dominant removal mechanisms. For further insight, assessment of the biomass elemental composition would be required at specific time points. Since both living and non-living algal biomass can uptake toxic elements [1,66], early biomass decomposition due to low pH and the presence of toxic elements should be also considered.

Overall, the combined evidence supports a mixed-mechanism framework in which rapid biosorption and ion exchange dominate early-stage removal, while biological uptake, stress responses, and potential desorption processes modulate longer-term behavior. These findings provide a basis for integrating bioremediation into mining wastewater treatment processes, discussed in the next section.

### 3.5. Bioremediation Strategies Targeting Mining Wastewater

**Figure 8:**
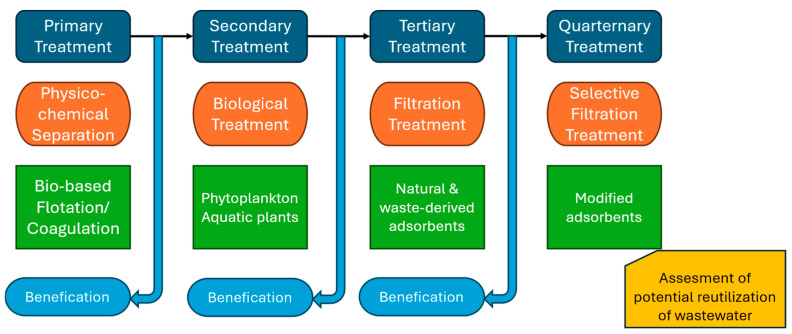
Potential integrative workflow targeting mine wastewater with bioremediation as secondary treatment.

This study suggests that single-strain treatments exhibit similar removal patterns across phytoplankton and perform comparably to *Chlorella vulgaris* and *Salvina natans*, indicating that a consortium is not necessary. However, the regulated multielement screening reveals clear constraints in bioremediation efficiency for Li and Se removal. Thus, absorbent-based treatment may serve as a complementary solution to target remaining elements (Avsar et al., in prep., [42]). For a broader removal of both regulated and unregulated elements at concentrations of up to 50 µg/L, as well as organic flotation reagents, all tested species proved to be effective. Nevertheless, individual species can have much higher bioremediation capabilities towards specific contaminants under optimized boundary conditions (e.g., pH, ionic speciation, and nutrient availability). The clear advantages of bioremediation are largely attributed to the minimal chemical input required for its application [67,68]. Screening of the mine outflow demonstrated higher removal capacities than those observed in synthetic wastewaters. The more complex composition of the mine outflow suggests a stronger species-dependent removal performance than that observed in the synthetic wastewater. All treatments (two synthetic wastewaters containing regulated and unregulated elements, as well as mine outflow) had a complex composition. Their effects were evaluated using the aquatic plant *Salvinia natans*, with a focus on photosynthetic pigment content to assess species sensitivity, as changes in these pigments are a common indicator of plant stress. The obtained results suggest that *Salvinia natans* seems to be less sensitive to unregulated elements, whereas exposure to regulated elements in synthetic wastewater induced negative effects, likely attributable to toxic elements such as As, Cd, Cr, Pb, and U. Accordingly, this study provides insight into the existing knowledge gap regarding bioremediation performance in natural versus synthetic wastewater systems [69]. *Chlorella vulgaris* demonstrated broader and more efficient overall removal of elements compared to *Salvina natans*. These results suggest that more diverse biological consortia could further improve remediation performance. Previous studies have also shown that biological consortia can effectively target a wide range of organic and inorganic pollutants [70,71]. The self-sustaining nature of bioremediation can be further optimized through nutrient supplementation, as phytoplankton and aquatic plants require micro- and macronutrients, particularly nitrogen and phosphorus [72,73]. For the treatment of mining wastewater, these aspects can be implemented within a multi-stage approach, integrating initial physiochemical separation, followed by biological treatment, and subsequent stages of targeted adsorbent-based filtration (Figure 8). In the case of biosorption, elements of interest may also be desorbed and recovered in concentrated form as by-products [74].

These findings support a structured treatment in which bioremediation serves as a low-chemical secondary method for general contaminant removal, complemented by selective adsorbents or other polishing technologies for more challenging targets such as Se and Li. The proposed workflow therefore provides a transferable screening-to-implementation pathway for selecting organisms and defining treatment windows under both controlled and environmentally relevant water matrix conditions.

## 4. Materials and Methods

### 4.1. Species Selection

Phytoplankton species, including algae and cyanobacteria, along with an aquatic fern, known for their ability to remove regulated and unregulated elements as well as organic flotation reagents, were selected for this study. The phytoplankton species were acquired from the Norwegian Institute for Water Research (NIVA) and incubated for 3 months under standard laboratory conditions prior to the incubation experiment (Table 2). This selection strategy ensured representation of diverse functional groups (green algae, cyanobacteria, and a macrophyte) with documented metal binding and uptake capacities, thereby enabling cross-taxa comparability under harmonized screening conditions.

### 4.2. Pollutants Selection

The removal efficiency of the selected phytoplankton and plant species was tested for regulated elements, as defined by Drinking Water Directive (EU) 2020/2184, as well as for unregulated elements and organic flotation reagents (Table 3).

For the exposure experiments, synthetic wastewater containing 50 µg/L of either regulated or unregulated elements was prepared in ultrapure water using analytical-grade multielement stock solutions (PerkinElmer Multielement Calibration Standard 2 and 3, respectively). The concentration of 50 µg/L encompasses threshold levels for regulated elements according to EU legislation while avoiding analytical and physiological artifacts associated with higher concentrations. The synthetic organic flotation reagent solution was prepared by mixing EDTA and PA at initial concentrations of 100 mg/L, and QA and OHA at 10 mg/L, using commercially available reference standards. The initial concentration of the four eco-friendly organic flotation agents was selected based on their corresponding analytical detection limits. Grouping contaminants into regulated elements, unregulated elements/REEs, and flotation reagents allowed the experiments to address both current compliance targets (e.g., Drinking Water Directive parameters) and emerging risk drivers associated with critical raw materials and modern beneficiation practices.

### 4.3. Exposure Experiments

#### 4.3.1. Phytoplankton Exposure to Synthetic Wastewater

Seven-day batch experiments were conducted using three synthetic wastewater formulations, each containing either regulated elements, unregulated elements (prepared from Multielement Calibration Standard 2 and 3 (PerkinElmer, Waltham, MA, USA)) or a mixture of organic flotation reagents (prepared from commercially available standards purchased from Merck, Darmstadt, Germany), alongside ultrapure water controls. Experiments were conducted under 12 h day and night cycles with photosynthetic flux density at 38 μmol/m^2^/s and stimulation using red (650 nm) and blue (455 nm) wavelengths to simulate natural environmental conditions [88,89]. The homogenous distribution was maintained using an orbital shaker at 75 rpm. Each treatment consisted of 100 mL serum vials containing 35 mL of synthetic wastewater and 5 mL of phytoplankton inoculum prepared in BG-11 nutrient solution. The control contained 35 mL ultrapure water and 5 mL phytoplankton-containing nutrient solutions. Each species was tested against all three contaminant groups along with its corresponding control pair to assess interspecific variability. At predefined time intervals, aliquots of 1 mL were collected from both treatment and control vials, filtered through 0.45 μm cellulose acetate membrane syringe filters (Isolab, Eschau, Germany), and individually prepared for analyses.

#### 4.3.2. Salvinia Exposure to Synthetic Wastewater

*Salvinia natans* was purchased from a local supplier and propagated vegetatively in tap water under ambient conditions (19–23 °C, natural photoperiod) to produce a stock culture. Individuals with a growth age of 30–40 days were selected for experimental use to ensure physiological uniformity. Exposure experiments were conducted using 2 synthetic wastewater formulations containing either regulated or unregulated elements at 50 µg/L concentrations. Control treatments consisted of modified Hoagland nutrient solutions prepared according to Torok et al. (2022) [18,90].

Each experimental pot (11.5 cm diameter) contained 2 g ± 0.04 g fresh weight (FW) of *Salvinia natans* in 200 mL of artificial wastewater. Experiments were conducted in triplicate over a 7-day period at room temperature (19–23 °C) under natural light conditions in order to keep the experimental set-up as simple as possible. According to previous observations, *Salvinia natans* showed good performance under moderate to high, as well as natural, light conditions; therefore, natural illumination was considered appropriate for this study. The experimental pots were kept near the window, where the light intensity varied depending on ambient conditions (sunny/rainy days, with higher or lower light intensity). Overall, the light regime corresponded to early to mid-spring conditions, during the experimental period, with an estimated light intensity ranging from ~20 to 150 µmol/m^2^/s. Following exposure, water samples and plant material were collected for analysis. The harvested plant material was rinsed with distilled water, oven-dried at 50 °C, and ground prior to analysis. Water samples were collected at defined time intervals (0, 3, 6, 12, 24, 36, 48, 60, 72, 96, 120, 140, and 168 h).

Physiological stress response was evaluated by quantifying the content of photosynthetic pigments: chlorophyll a (Chla), chlorophyll b (Chlb), and total carotenoids (Car). Pigments were extracted from 0.4 g fresh plant tissue using 5 mL of 100% (*v*/*v*) methanol. Cell disruption was facilitated by sonication for 15 min, followed by incubation at 70 °C for 5 min to enhance the extraction. The extracts were centrifuged to separate cellular debris, and the supernatant absorbance was measured at 470, 652, and 665 nm using a NanoDrop One UV–VIS Spectrophotometer (Thermo Fisher Scientific, Waltham, MA, USA). Pigment concentrations (Chla, Chlb, and Car) were calculated according to Lichtenthaler [91] and expressed as µg/g FW.

#### 4.3.3. Exposure to Mine Outflow

There are several underground mining operations located in the southern part of the Igniș Massif, near the Băița locality in Maramureș County, northwestern Romania. The region has a long history of mining Neogene hydrothermal polymetallic deposits, primarily containing Pb, Zn, Cu, Au, and Ag. After mine closure, the outflow of the mine galleries is received by the Băița River, that subsequently drains into the Lăpuș River. A volume of 5 L mine outflow water (pH = 3.2) was collected from one of the closed mine galleries in pre-cleaned polyethylene bottles and kept at 4 °C until analysis. For element analysis the samples were filtered through 0.45 µm cellulose acetate membrane syringe filters and acidified with 65% HNO_3_ to pH < 2). The elemental removal efficiency of *Chlorella vulgaris* and *Salvinia natans* was assessed in 7-day batch experiments. The elemental concentrations of the outflow water were measured before (day 0) and after the treatments (day 7).

### 4.4. Chemical Analysis

#### 4.4.1. Elemental Analysis

Quantification of metals was conducted using an iCAP TQ inductively coupled plasma mass spectrometer (ICP-MS) equipped with a triple-quadrupole mass filter (Thermo Fisher Scientific, Waltham, MA, USA) controlled via Qtegra software (v. 2.10.3324.131) for the experiments involving aquatic plants and using an inductively coupled plasma tandem mass spectrometry (ICP-MS/MS, Thermo Fisher Scientific, Waltham, MA, USA) for the experiments using phytoplankton species.

A quantity of 200 mg (dry weight, DW) of control and treated plant sample was digested with 5 mL of 65% HNO_3_ (Merck, Darmstadt, Germany) in a closed-vessel microwave digestion system (Speedwave XPERT, Berghof, Eningen, Germany) and diluted with ultrapure water to a final volume of 25 mL. The analyzed results were expressed in mg/kg DW.

The elemental concentrations of As, Se, Ag, V, Pb, Ti, Cu, Cr, Cd, Co, Zn, Be, Ni, Ba, Sc, Tb, Dy, Gd, Ho, Pr, Tm, Er, Lu, Sm, Eu, Yb, Nd, Ce, Y, and La as well as the naturally occurring radionuclides (NORs) such as ^238^U, ^232^Th, and ^226^Ra were measured in the acidified aqueous samples of the phytoplankton experiments using an iCAP MTX-900 instrument (Thermo Fisher Scientific, Waltham, MA, USA). The method configuration included He and O_2_ as collision gases and the kinetic energy discrimination (KED) mode. Calibration was performed using single-element ICP standard solutions (Inorganic Ventures^®^, Christiansburg, VA, USA), with Bi and Rh as internal standards. Measurement accuracy was verified using standard reference materials (CRMs) including multi-analyte solutions IV-ICPMS-71A and CMS-1 (Inorganic Ventures^®^, Christiansburg, VA, USA) for stable elements, ^238^U, and ^232^Th, and digested IAEA-448 (^226^Ra in soil from oilfield) for ^226^Ra. Furthermore, detection limits were established from blank measurements.

#### 4.4.2. Quantification of Organic Flotation Residuals

UPLC-HRMS (ultrahigh performance liquid chromatography coupled to high resolution mass spectrometry) was used to quantify the organic flotation residuals. The analysis was performed using a UPLC (Ultimate 3000 chromatograph with autosampler) coupled to a QExactive detector from Thermo Fisher Scientific (Waltham, MA, USA). The MS was operated in electrospray ionization (ESI) interface in negative ionization mode. The UPLC method was applied with modifications following Jaén-Gil et al. (2019) [92]. Chromatographic separation was performed using an Acquity BEH C18 column (150 mm × 2.1 mm i.d. 1.7 μm particle size; Waters Corp. Milford, MA, USA) using a binary mobile phase at 0.25 mL/min, with a total run time of 23 min. For negative ion mode, solvent A was a NH_3_ aqueous solution (pH = 9), and solvent B (methanol). The gradient elution started at 95% A, increased to 100% B over 16 min, and returned to 95% A at 23 min. Solvent A decreased to 85% at 8 min at a linear rate of 2.5, then decreased to 0% at 16 min at a linear rate of 21.25. Solvent A was held at 0% for 4 min, then increased to 85% at 14 min at a linear rate of 42.5, and finally to 95% at 15 min at a linear rate of 10. The flow rate was maintained at 0.25 mL/min. A 10 μL aliquot of each sample was directly injected into the system. For MS detection, a full-scan acquisition method was applied at 70,000 resolution power over a mass range of 50–750 Da to maximize data collection. In parallel, data-dependent acquisition (DDA) was employed, where ions exceeding a threshold of 1000 counts triggered MS/MS scans at 35,000 resolution. Organic flotation residuals were identified and quantified using diagnostic mass fragments: PA([M-] = 658.85409 *m*/*z*), EDTA ([M-] = 291.08338 *m*/*z*), QA ([M-] = 191.05611 *m*/*z*), OHA ([M-] = 158.11865 *m*/*z*). For each organic flotation agent, a calibration curve was established, yielding detection limits of 50 µg/L for QA and OHA, and 500 µg/L for PA and EDTA.

### 4.5. Data Analysis

The removal efficiencies (%) of the unregulated and regulated elements, as well as the organic flotation reagents, were calculated using Equation (1):

(1) $$E=\frac{C_{i}-C_{f}}{C_{i}}\times 100$$

where E (%) represents the percentage removal efficiency (%), C_i_ is the initial concentration of the individual contaminant (mg/L) in the wastewater prior to treatment, and C_f_ is the residual metal concentration in the wastewater after treatment (mg/L). For the experiments, C_f_ corresponds to the contaminant concentration in the control sample at the same time point, to account for element release from the phytoplankton and aquatic plant cells.

For plant experiments, statistical analyses were performed on four independent biological replicates (mean ± SD, n = 4). Differences between treatments were evaluated using Tukey’s HSD post hoc test at a significance level of *p* ≤ 0.05, performed in OriginPro software (version 2020b).

## 5. Conclusions

This screening study demonstrates that both phytoremediation and phycoremediation are effective nature-based solutions for removing a wide range of regulated and emerging contaminants commonly found in mining wastewater. The combination of 10 phytoplankton and an aquatic fern that were screened in successive multielement and emerging substance solutions provides some novel insights. Across all standardized screening experiments, all phytoplankton species and *Salvinia natans*, the aquatic fern, exhibited high removal efficiencies for key elements, including Pb, Ag, U, and several REEs under acidic, multielement environments. Time-resolved analyses revealed rapid removal for numerous contaminants, especially REEs, highlighting significant bioremediation capabilities within the first 24 h. While removal efficiencies remained high for most contaminants, Li and Se represented notable limitations, likely due to competitive interactions with major cations and physiological constraints. Based on photosynthetic pigment responses, *Salvinia natans* showed lower sensitivity to unregulated elements, whereas exposure to regulated elements in synthetic wastewater induced measurable but moderate stress effects. The comparison between synthetic and real wastewater further confirmed that simplified matrices may underestimate bioremediation performance in complex environmental waters. These findings collectively indicate that selected microalgal species, particularly *Chlorella vulgaris*, and *Salvinia natans*, possess strong potential for treating mine-affected waters, while also offering opportunities for optimizing metal recovery and resource valorization. However, the observed contaminant-specific variability highlights the importance of adapting species selection to site-specific wastewater matrices. This study was constrained in its screening framework to provide more mechanistic insights explaining the observed removal trends. To advance these bioremediation solutions toward real-world application, future studies should expand the experimental framework to include variable pH regimes, higher contaminant concentrations, and larger wastewater volumes, alongside increased replication to enhance statistical power and reproducibility. Simultaneously, it is crucial to evaluate long term performance and biomass stability under sustained exposure, as well as to validate effectiveness in real process water outflows, explicitly considering the differences observed between synthetic and environmental wastewater matrices. For organic flotation reagents, follow-up studies should go beyond parent compound removal to characterize transformation products, ensuring that treatment reduces overall environmental risk. Finally, evaluating mixed microbial, algal, and plant consortia for potential synergistic effects, and integrating bioremediation as a secondary or polishing step within multi-stage mine water treatment systems, will help translate screening results into scalable, low chemical, and sustainable treatment strategies for complex mining effluents.

## Figures and Tables

**Table 1:** The elemental composition of the mine outflow water.

Element	Al	Mg	Fe	Zn	Mn	Cu	Pb	Sr	Ba	Cd	Co	Ni
Concentration (µg/L)	16,775	8201	6096	3560	1338	111	49.2	35.8	35.2	31.9	28.1	26.0
Parametric value (µg/L)	200 *	**	200 *	5000	50 *	2000	5	**	**	5	**	20
Element	Li	Rb	Cr	Ag	Cs	Tl	As	Be	U	Se	V	
Concentration (µg/L)	12.2	8.03	5.17	5.13	3.92	2.82	1.40	1.02	0.88	-	-	
Parametric value (µg/L)	**	**	25	**	**	**	10	**	30	10	**	

* Indicator parameters, DIRECTIVE (EU) 2020/2184 of the European Parliament and of the Council of 16 December 2020 on the quality of water intended for human consumption. ** Not regulated in EU Directive 2020/2184.

**Table 2:** Phytoplankton species selected for the screening experiments.

Species	Type	Removal Targets
*Chlorella vulgaris* [11,32,33]	Algae (green algae)	Pb^2+^, Cd^2+^, Cr^6+^, Cu^2+^, Fe^3+^, Hg^2+^, Ni^2+^, Th, U, Zn^2+^
*Nannochloropsis oculata* [75,76]	Algae (sea water)	Pb^2+^, Cd^2+^, Cu
*Botryococcus braunii* [77,78]	Algae (green algae)	As^3+/5+^, Zn^2+^
*Klebsormidium* sp. [79]	Algae (green algae)	Cd^2+^, As^3+^, Pb^2+^, Cu^2+^, Ni^2+^
*Chlamydomonas reinhardtii* [11]	Algae (green algae)	Cd^2+^, Co, Cr^6+^, Cu^2+^, Hg^2+^, Ni^2+^, Pb^2+^, Zn^2+^
*Spirogyra* sp. [11,80]	Algae (green algae)	Cd^2+^, Co, Cu^2+^, Hg^2+^, Ni^2+^, Pb^2+^, Zn^2+^
*Scytonema* sp. [81]	Cyanobacteria	Cd, Co, Cr, Ni, Zn
*Synechococcus elongatus* [82,83]	Cyanobacteria	Cu, Pb, Ni, Cd, Cr, U
*Spirulina* spp. [11]	Cyanobacteria	Cd^2+^, Co, Cr^3+^, Cr^6+^, Cu^2+^, Hg^2+^, Ni^2+^, Zn^2+^
*Anabaena torulosa* [84]	Cyanobacteria	Cr, U
*Salvinia natans* [22,85,86,87]	Aquatic fern (Polypodiopsida)	As, Cd, Cr, Cu, Hg, Ni, Pb, Zn

**Table 3:** Contaminant groups selected for the screening experiments.

Contaminant Group	Constituents
Regulated elements *	Ag, As, Be, Cd, Co, Cr, Cu, Mn, Ni, Pb, Se, Th, Tl, U, V
Unregulated elements	Ce, Dy, Er, Eu, Gd, Ho, La, Li, Lu, Nd, Pr, Sc, Sm, Tb, Tm, Y, Yb
Organic flotation reagents	Octanohydroxamic acid, quinic acid, EDTA, phytic acid

* Drinking Water Directive (EU) 2020/2184.

## Data Availability

The original contributions presented in this study are included in the article and Supplementary Material. Further inquiries can be directed to the corresponding authors.

## Supplementary Materials

The following supporting information can be downloaded at: https://www.mdpi.com/article/10.3390/molecules31091494/s1; Figure S1: Temporal variation of elements concentrations in synthetic water treated with *Chlorella vulgaris* (a) and *Salvinia natans* (b) during a 7-day exposure to regulated and unregulated elements; Table S1. Removal efficiencies (%) of the tested species in the 3 different artificial wastewater experiment: (a) regulated elements, (b) unregulated elements (Figure 1, in main manuscript), as well as (c) organic flotation reagents (Figure 2, in main manuscript).
