# Supplementary material for: Screening Bioremediation for the Effective Removal of Regulated and Emerging Contaminants from Mining Wastewater
Source: Molecules. 2026 Apr 30;31(9):1494. doi: 10.3390/molecules31091494 (PMC13164779; doi:10.3390/molecules31091494)
Supplement: Supplementary file 1 [file molecules-31-01494-s001.zip › molecules-4257795-supplementary.pdf]

molecules

MDPI

Supplementary Material

Screening Bioremediation for the Effective Removal of Regulated and Emerging Contaminants from Mining Wastewater

Niroshan Gajendra [1,*,†], Anamaria Iulia Török [2,*,†], Deniz Avsar [1], Mila Kristiina Pelkonen [3], Simion Bogdan Angyus [2], Ragne Lundeby Grønvold [3], Claudiu Tănăselia [2], Erika Andrea Levei [2] and Laura Ferrando-Climent [1]

[1] Department of Tracer Technology, Environmental Technology Section, Institute for Energy Technology, Instituttveien 18, 2007 Kjeller, Norway; deniz.avsar@ife.no (D.A.); laura.ferrando-climent@ife.no (L.F.-C.)

[2] Research Institute for Analytical Instrumentation Subsidiary, National Institute of Research and Development for Optoelectronics, Donath 67, 400293 Cluj-Napoca, Romania; bogdan.angyus@icia.ro (S.B.A.); claudiu.tanaselia@icia.ro (C.T.); erika.levei@icia.ro (E.A.L.)

[3] Department of Environmental Safety and Radiation Protection, Environmental Technology Section, Institute for Energy Technology, Instituttveien 18, 2007 Kjeller, Norway; mila.pelkonen@ife.no (M.K.P.); ragne.gronvold@ife.no (R.L.G.)

* Correspondence: niroshan.gajendra@ife.no (N.G.); iulia.torok@icia.ro (A.I.T.)

† These authors contributed equally to this work.

Academic Editor: Vasilios Sakkas

Received: 29 March 2026

Revised: 22 April 2026

Accepted: 24 April 2026

Published: 30 April 2026

Copyright: © 2026 by the authors. Submitted for possible open access publication under the terms and conditions of the Creative Commons Attribution (CC BY) license.

This online resource contains the following data:

Figure S1. Temporal variation of elements concentrations in synthetic water treated with *Chlorella vulgaris* (**a**) and *Salvinia natans* (**b**) during a 7-day exposure to regulated and unregulated elements

Table S1. Removal efficiencies (%) of the tested species in the 3 different artificial wastewater experiment: (a) regulated elements, (b) unregulated elements (Figure 1, in main manuscript); as well as (c) organic flotation reagents (Figure 2, in main manuscript)

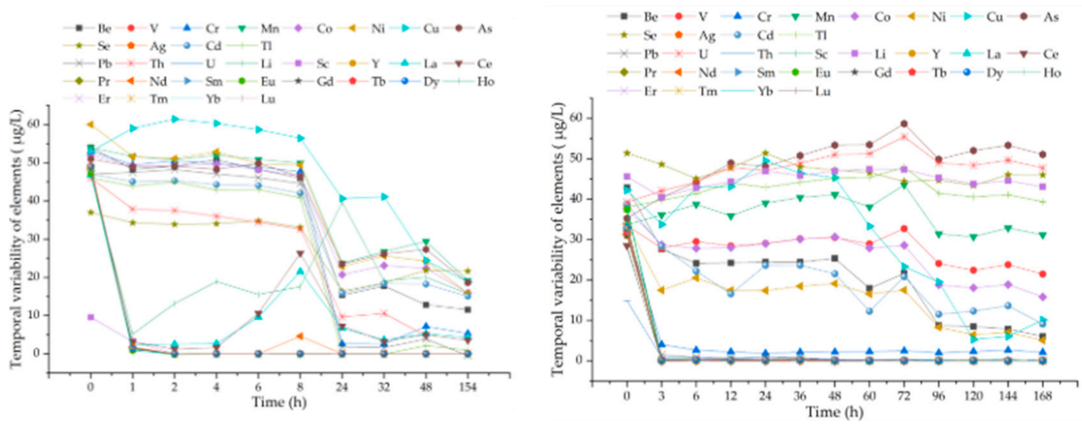

(**a**) (**b**)

Figure S1. Temporal variation of elements concentrations in synthetic water treated with *Chlorella vulgaris* (**a**) and *Salvinia natans (***b***)* during a 7-day exposure to regulated and unregulated elements

https://doi.org/10.3390/molecules31091494

Table S1. Removal efficiencies (%) of the tested species in the 3 different artificial wastewater experiment: (a) regulated elements, (b) unregulated elements (Figure 1, in main manuscript); as well as (c) organic flotation reagents (Figure 2, in main manuscript)

(a) Regulated elements – removal efficiencies (%)															
**Species**	**Se**	**Mn**	**Cu**	**As**	**Cd**	**Co**	**V**	**Ni**	**Be**	**U**	**Cr**	**Tl**	**Th**	**Ag**	**Pb**
Scytonema sp.	39,1	67,7	73,2	67,7	71,8	72,2	69,6	75,7	81,1	84,3	91,4	96,4	100	99,6	101
Spirogyra sp.	40,6	62,4	76,2	63,4	69,3	68,8	74,1	73,6	82,0	84,0	92,3	97,2	98,1	99,6	100
Anabaena torulosa	41,4	64,5	64,5	63,5	68,0	69,3	70,8	73,6	78,6	80,0	89,9	97,9	89,7	99,6	101
Chlorella vulgaris	31,2	59,2	62,7	58,7	65,0	64,4	69,0	70,1	77,9	94,6	91,4	99,7	99,8	100	99,5
Klebsormidium sp.	32,5	56,4	67,5	58,7	63,0	65,3	66,3	68,6	76,9	78,9	92,4	99,1	99,6	99,6	100
Synechococcus elongatus	34,8	59,3	61,0	59,7	62,2	65,6	67,1	70,9	75,4	77,3	93,4	98,8	100	99,6	100
Botryococcus braunii	25,7	53,4	47,0	55,9	55,4	60,8	57,9	66,7	67,6	68,3	91,5	100	99,1	99,6	100
Chlamydomonas reinhardtii	19,9	55,6	49,0	57,5	56,1	61,5	60,9	67,1	67,6	74,9	86,4	99,4	99,9	99,6	100
Nannochloropsis oculata	36,8	53,8	57,2	58,9	56,3	61,0	57,9	66,5	73,0	74,1	68,2	89,9	90,6	87,1	92,4
Spirulina major	22,0	45,1	36,3	53,0	49,3	49,1	54,9	62,3	60,4	64,7	86,9	100	100	99,6	101
Salvinia natans	37,0	n.d.	37,0	13,0	44,0	17,0	97,0	33,0	35,0	98,0	91,0	n.d.	97,3	96,0	99,0

https://doi.org/10.3390/molecules31091494

(b) Unregulated Elements – removal efficiencies (%)																	
**Species**	**Li**	**La**	**Ce**	**Tb**	**Dy**	**Ho**	**Er**	**Tm**	**Yb**	**Lu**	**Y**	**Gd**	**Eu**	**Sc**	**Sm**	**Pr**	**Nd**
Scytonema sp.	37,1	59,4	53,8	100	100	99,7	100	100	100	100	99,7	100	100	100	100	102	94,6
Spirogyra sp.	28,8	103	110	100	100	99,7	100	100	100	100	99,7	100	100	100	100	101	103
Anabaena torulosa	25,9	83,6	86,2	100	100	99,7	100	100	100	100	99,7	100	100	100	100	100	101
Chlorella vulgaris	30,3	92,5	96,7	100	100	99,7	100	100	100	100	99,7	100	100	100	100	100	100
Klebsormidium sp.	26,8	76,2	79,1	100	100	99,7	100	100	100	100	99,8	100	100	100	100	102	105
Synechococcus elongatus	41,7	78,7	80,4	100	100	99,7	100	100	100	100	99,7	100	100	100	100	101	102
Botryococcus braunii	45,1	97,2	103	100	100	99,7	100	100	100	100	99,7	100	100	100	100	100	101
Chlamydomonas reinhardtii	28,2	93,4	99,9	100	100	99,7	100	100	100	100	99,7	100	100	100	100	101	102
Nannochloropsis oculata	42,7	73,8	76,7	100	100	99,7	100	100	100	100	99,8	100	100	101	100	100	101
Spirulina major	21,9	84,0	87,9	100	100	99,7	100	100	100	100	99,7	100	100	100	100	100	101
Salvinia natans	0,0	99,9	100	99,8	99,8	99,8	99,8	99,8	99,8	99,8	99,8	99,8	99,8	99,2	99,8	99,9	99,9

https://doi.org/10.3390/molecules31091494

**(c) Organic Flotation Reagents – removal efficiencies (%)**				
**Species**	QA [10 mg/l]	EDTA [100 mg/l]	PA [100 mg/l]	OHA [10 mg/l]
Nannochloropsis oculata	97,5	99,8	102	100
Botryococcus braunii	100	81,7	110	100
Spirogyra sp.	87,1	92,2	102	100
Synechococcus elongatus	84,6	91,9	104	100
Spirulina major	85,8	92,2	100	100
Chlorella vulgaris	86,7	92,2	96,2	100
Klebsormidium sp.	84,9	92,1	98,0	100
Chlamydomonas reinhardtii	86,4	92,5	92,2	100
Anabaena torulosa	82,9	91,9	90,7	103
Scytonema sp.	83,7	92,1	91,3	100

https://doi.org/10.3390/molecules31091494
